# Ten simple rules for acing virtual interviews

**DOI:** 10.1371/journal.pcbi.1009057

**Published:** 2021-06-24

**Authors:** Dalen Chan, Christina M. Fitzsimmons, Mariana D. Mandler, Pedro J. Batista

**Affiliations:** Laboratory of Cell Biology, National Cancer Institute, National Institutes of Health Bethesda, Maryland, United States of America; Dassault Systemes BIOVIA, UNITED STATES

## Introduction

Interviews are commonly required at every stage of the academic journey from undergraduate admission to prospective faculty hiring. For applicants, the on-site interview is an important opportunity not only to meet future peers, colleagues, and mentors, but also to learn more about the culture of the university and the surrounding neighborhood where they may be spending the next few years of their lives. However, in light of the current Coronavirus Disease 2019 (COVID-19) pandemic, a number of programs have announced plans to eliminate in-person interviews and instead have virtual interviews [1–4]. Popular video platforms, including Gather, Webex, and Zoom, offer an alternative to the traditional in-person interviews during times of social distancing and university-wide travel restrictions. However, while these formats may be familiar for participating in classes or staying connected with friends, many young scientists are less experienced with virtual school or job interviews. Here, we offer a list of 10 simple rules to assist early-stage scientists with virtual interviews in an academic or scientific setting.

## Rule 1: Keep it professional

The most important thing is to treat the interview professionally. When communicating with schools, use an email address that is checked regularly. Whether this email is affiliated with a school, work, or personal account is a matter of individual preference. However, the account that is used should have a professional username (e.g., LastName_FirstName [at] university [dot] edu). Avoid using email addresses that are suggestive or joking. The same advice holds true for video conferencing platforms. It is best to use an account with a professional screen name and a recent, clear headshot.

A professional social media account can help to establish a positive online presence even before the interview. Since many admission committees and potential mentors will search for applicants’ social media profiles, it is good to be mindful of damaging, inappropriate content. A post that is funny to one person may be interpreted in different ways by different audiences. One recommendation is to have both a professional and a private social media account and to clean up any old, unused accounts. Depending upon your career stage, it may be helpful to create a professional-looking web page to highlight your accomplishments and build a personal brand. There are many free or low-cost platforms that let people create a website without the need for much programming experience [5].

Along with a professional online presence, you should plan to “dress for success” [6]. While a virtual interview allows you to choose comfortable attire options, it is important to show you have a certain degree of respect for the interviewer. If, for example, a program is known to be very formal, this may mean a jacket and tie or pants suit. However, if the program is more relaxed, a business casual sweater, button-down shirt/blouse, or a professional-looking dress can be substituted for a suit. Equally important, do not rely on the fact that the camera only shows your upper body. You may need to stand up or walk to a different room during the interview, so it is important to dress from head to toe. In some cases, graduate programs recognize that it is often your first experience with professional interviews, and they will recommend a dress code. Use this as a helpful guide when deciding what to wear, and if you are still in doubt, ask a trusted advisor or mentor. Asking about this will not reflect negatively. Ultimately, it is important to wear attire that is suitable but also makes you feel comfortable, so you can relax and concentrate on the task at hand.

## Rule 2: Test the technology

Before the day of the interview, it is important to test and familiarize yourself with the technology that will be used. Download and install any programs or apps that may be required (e.g., Zoom, Webex, etc.), and try a practice call with a friend or colleague to check the sound and video connection. One common audio problem is static or background noise. A simple pair of earbuds or headphones can be used to reduce the noise and improve sound quality. In addition to testing the audio and video, ensure that the default screen name associated with the account is professional (Rule 1). When possible, it is strongly recommended that an Ethernet connection is used instead of a wireless connection. Using a wired connection can also improve problems with lagging or freezing video. If you are using a wireless connection, try to avoid free or guest networks if possible, as the connection may lag or be unsecured. Sometimes, unforeseen difficulties arise (Rule 7). We all run into issues with technology, and technical problems are often easily forgiven. Remaining calm and working through the problem is a chance to demonstrate how you handle unexpected challenges or stress.

## Rule 3: Pick a good location, and prepare the environment

In addition to preparing technology, preparing your environment can help ensure a smooth process on the day of the interview. As the old adage goes, “Location! Location! Location!” Location is important to ensure an interview free of distractions for both you and the interviewer, so that you can give it your full focus and attention. Take the time to identify a quiet space with good acoustics. For some, this might be a personal bedroom, living area, or even a large closet. For others, this space may be a conference room or office in the lab. In some cases, you might ask your advisor to help you locate a quiet space for the interview.

Many trainees may not have the option of leaving home for an interview. If you live with roommates, children, or even pets, it is important to develop a plan. Inform your roommates or family members of your interview in advance. On the morning of the interview, remind them again, and hang a sign on the door, indicating that you should not be disturbed. For those with children or pets, one option might be to have a trusted family member or friend watch them. If this is not an option, inform your interviewers at the beginning of any situations that may require your attention. If you encounter difficulty identifying the perfect location, you can use technology to your advantage, such as headphones with built-in noise reduction that are designed for gaming or making phone calls.

Once your quiet space has been identified, there are a few additional items that you can prepare to ensure a smooth interview. It is important to check the lighting in the room. Changing the lighting angle or the position of your desk with regard to the lighting can help you avoid strange light halos around your head or screen glare if you wear glasses. Along with the lighting, be sure your camera view is free of clutter. If you are unable to change the camera angle, many video platforms offer the ability to use a blurred or virtual background. If you decide to use one of these options, be sure to test it when you test your technology. As in Rule 1, make sure the virtual background you select is appropriate. Be aware of where the camera is pointing so you can make eye contact with your interviewer, especially if you have multiple screens. It can be helpful to use books or other materials to elevate your computer so that your camera naturally sits at eye level. Most virtual platforms allow you to rearrange the layout of the windows. By moving the video of the interviewer directly beneath the camera, it gives the appearance that you are looking directly at the interviewer. Additionally, many platforms provide a self-view that will allow you to see how you appear on camera. However, be mindful that looking at yourself may be distracting. If you notice you are looking at yourself too frequently, many video platforms allow you the option of hiding this view so you can more easily focus on the other person.

Finally, make yourself and your interview location as comfortable as possible, and think of any items you might need. This could include a pillow for your chair, a glass of water, tissues, a notebook, pens and pencils, a phone charger, or other small items you might need throughout the course of the interview. Check that all technology (laptop, headphones, etc.) are plugged in and at full charge. Lastly, silence your phone, and close any applications or websites with pop-up notifications and distracting sounds. An advantage of a virtual interview is that you have some control over the setting for your interview. Making it as comfortable as possible will allow you to reduce some of the stress associated with the process and show your authentic self.

## Rule 4: Be an interviewer AND an interviewee

When you plan an interview with someone, it can be very helpful to take the perspective that you are both an interviewee and an interviewer. This may actually take some of the stress out of the interview, since both you and the person with whom you are speaking are learning about each other. As such, there are a few very important ways to ensure a smooth interview, especially in a virtual setting.

One of the best ways to ensure a smooth interaction is to have a solid introduction of yourself and your research prepared ahead of time. This can help you prepare for common opening questions such as “Tell me about yourself,” and “Why are you interested in this position?” The person speaking with you is trying to select someone they will be excited to have as a new colleague. Therefore, you want to convey your identity as a scientist and show that you have thought about your research. It can be helpful to make two to three broad overview slides describing your past research topic(s), findings, and implications. Make sure you know ahead of time how to share the slides you prepared with the video application you plan to use. It is important to practice mock interviews and practice talks with peers and mentors. As with in-person interviews, you should be prepared to answer questions about your past research, as well as the topics you are interested in studying in the future [7]. You may decide to take short handwritten or typed notes during the interview (these can be helpful for a follow-up email; Rule 9). Be aware your interviewer is likely to take the cue that you are taking notes more readily if you are briefly looking down from the camera. Consider taking notes or recording impressions between interviews to avoid unnecessary distractions.

Second, as with in-person interviews, it is crucial that you learn about the interviewer beforehand. Often, a schedule will be shared with you ahead of the interview so you know which people you will be meeting. Take the opportunity to learn about the individuals before you begin the interviews. Faculty websites can be a great starting point to learn more about the research directions of a lab, providing an opportunity to connect with students in your field of interest. However, keep in mind that not all professors update their websites regularly. Often, the best way to learn about current research directions is by reading recent research papers published by the lab. Familiarizing yourself with recent publications and knowing the accomplishments of your interviewer will naturally make you a better conversationalist as you will more easily be able to ask intelligent questions or discuss specific research ideas. In many graduate school interviews, you may interview with an older student or current members of a research group. Take this as an opportunity to ask questions about topics such as teaching classes, living arrangements, or why the older student selected this program. While this portion of the interview may feel less formal, it can give you some important information.

Finally, it is important to have questions of your own prepared ahead of time. Remember, you are also interviewing the other person. These questions should be specific and not something that is easy to find on the school website. Particularly, when you may not be able to visit the campus in person, it is important to ask all of the questions that may be important to you, as it will help you make an informed decision. Bringing a list of questions to interviews is common and ensures that you cover all the topics that you intended. Table 1 contains examples of questions you may be asked or that you could ask current students or professors. As everyone has a different perspective, it can be beneficial to ask multiple people (professors, students, etc.) the same types of questions and compare the answers you receive.

**Table 1:** Questions that may be asked of you or that you can ask interviewers.

**Questions directed to you**
Why are you interested in joining our lab/our graduate program?
What areas of research are you most interested in, and why?
Tell us about your past research.
Tell us about a time you experienced a setback.
**Questions you might ask current students**
What is the average tenure of students in the lab?
Why did you decide to join this lab/graduate program?
How often do you meet with your advisor? How available are they when you need help on your projects? Are other students/neighboring labs available for help?
What sort of activities do you do for fun?
**Questions you might ask the faculty or program**
What have previous trainees from the lab/graduate program gone on to do?
How often do you meet with students?
What are the requirements to graduate from the lab/program?
What type of academic, financial, or other support is provided for students?

## Rule 5: Keep a familiar routine, and pace yourself

On the day of the interview, it is important to stick to your normal routine as much as possible. If you usually go for a run in the mornings, go out for a run. If you listen to the news while you eat breakfast, do that as well. If you do not usually drink coffee, today is not the day to start. Small adjustments to heighten your focus during an interview can help. If you have a personal method for warming up before stressful situations, such as listening to your favorite song or meditating, then plan to do so. Practice self-awareness to determine what you need to feel your most comfortable so you can be your most presentable and authentic self. One benefit of the virtual interview is you have control over your environment, without the stress of travel to an unfamiliar setting.

You may be interviewing with multiple people at one institution. Keep a copy of your itinerary and a notebook easily accessible so that you can refer to it throughout the day. Your itinerary may have a series of links to connect to the specific meetings, or you may stay in one “meeting room” while the interviewers enter and exit throughout the day. Between interviews, quickly skim your notes to refresh your memory of the person you will be speaking with, as well as any specific questions you had for that person. Remember, you are both interviewing each other!

Additionally, it is important to take a break between interviews. Although the itinerary might not allow for long breaks, if you are feeling stiff after a long period of sitting, try to find a couple of minutes to perform some simple stretches. Even something as simple as walking to a different room can give your muscles a chance to move and allow your eyes a break from the screen. If there is time allotted for longer breaks, try to plan snacks or meals to align with your schedule. It’s better to stick with familiar foods and not eat or drink anything that you would not normally consume.

## Rule 6: Communicate clearly, and ask for clarification

In virtual interviews, it can sometimes be difficult to know when another person is done speaking. If a question is asked of you, take a moment to pause to ensure that the other person has completed their thought before you begin to answer. When communicating, it can be beneficial to engage in body language to show interest during your interview. However, distractions such as pen tapping or fidgeting may take attention away from you and what you’re saying [6]. If you know that you are prone to such activities, try placing your hands on your lap or the armrest of your chair where they will not be seen unless necessary [8]. Sometimes, technical questions are best answered by drawing on a white board, which may not be straightforward during a virtual interview. You may be able to use specialized software that allows you to share a screen and draw a concept in real time. These software options are becoming more widely available and evolving as virtual interviews continue. If these options are not available, you could also attempt to position a white board in the view of the camera. As in Rule 2, practice with a friend or labmate before the interview to ensure full visibility. Similarly, if you are uncertain about certain aspects of the interview itself, do not hesitate to seek clarification. It is better to ask questions than to guess at what the other person wants or expects. In some cases, you may be assigned a point of contact for the interview. This could be another student, an administrator, or even the professor. The goal of the contact person is to help you have a smooth interview experience. If you have any questions about the itinerary, the format for the interview, or are having technical issues, do not hesitate to ask. If for whatever reason the contact person is busy, others are usually more than happy to help you.

## Rule 7: Embrace Murphy’s law: “Whatever can go wrong, will go wrong”

Sometimes, despite your best efforts to prepare, things go wrong. A powerful thunderstorm might knock out the power on your block. The internet may be slow because two people in your apartment have an interview on the same day. Or perhaps your neighbor has decided that this is the perfect time to mow the lawn right outside your window. For these reasons, it is important to stay calm, and accept that not everything will go as intended. Adjusting to an alternate pace shows that you are comfortable with difficult situations. However, preparing a backup plan for common scenarios can alleviate the stress of some of these unforeseen challenges.

If you are experiencing audio/visual disruptions such as being unable to hear or see the other speaker, communicate these challenges promptly. You many need to try exiting the platform and reentering the interview. If the internet is lagging, try turning off your video and just using the audio. If there is a lot of background noise, try moving to a different part of the house or lab. As a last resort, you may ask your interviewer to call back at a point later in the day. In a worst-case scenario, your computer may crash. In such extreme circumstances, having a phone number (written on a piece of paper) for your contacts at the university can help you navigate the crisis promptly. Try to keep calm and have a backup plan.

Attempt to be flexible with the timing of the interview. Either you or the interviewer may be running late due to a previous interview. It is also important to be aware of potential time differences. If you are ever uncertain, don’t hesitate to email your contact to clarify the time of the meeting. Above all, keep in mind that we’re all human. There will be user errors, tech glitches, and children singing songs in the background. Patience and grace with yourself and those you are interviewing with will go a long way toward having a positive experience.

## Rule 8: Connect with current students and the university environment

One aspect of virtual interviews that can be challenging is connecting with current students and learning more about the culture of the program, university, or city. It is important to take advantage of opportunities presented by the program to help you connect.

You might be able to take advantage of messaging platforms designed for teams and work groups. Some graduate departments have organized accounts in platforms such as Slack [1,9] where you can meet in small groups with current students or take virtual tours of the school. There may be channels within the account of the graduate program dedicated to topics such as program requirements or social life in the city. Additionally, some programs may offer an “informal” or “social hour” type of event at the end of the interview. Take advantage of these platforms to interact with current students, but do not feel pressured to stay the entire time if you are tired or need a break.

If the graduate program does not have an account on a messaging platform, search the university website to see if there are virtual tours offered, or ask a current student to give you an informal video tour of the lab or building via video. In situations like these, leverage your network of friends and friends of friends to connect with current students in the program or lab. Many current students or lab members are happy to take a short break from the lab to talk with you and answer questions.

In both cases, try to talk with a number of different people. A senior graduate student or postdoc in the lab may have a different perspective than someone who is in his or her first year. Additionally, pay attention to which students are attending these types of events. Do you feel that it reflects the composition of the program? Or do you find that you are always talking with the same three or four students? Be wary of graduate programs or labs that restrict your access to talking with other trainees.

## Rule 9: Send follow-up emails

After any type of interview, it is polite to send follow-up emails. It is not necessary to send an email to every single person with whom you interacted. However, if you had a particularly nice conversation, or someone went out of their way to help you, it is a good idea to send a short email expressing your gratitude. In general, it is considered good practice to thank the head of the interviewing committee, as well as every faculty member you met one on one.

Although it may be hard to establish a meaningful connection with your interviewers in a virtual setting, good note-taking skills during the interview could provide an avenue for you to write a thoughtful, personal response. When sending follow-up emails, try to mention an interesting tidbit about your conversation, but keep the message brief. This could be a project that you are interested in pursuing or ideas that raise new questions in that particular field. It is important to stay engaged even through this last part of the interview and continuing the conversation in this way will let the interviewer know that you are actively thinking about their work.

The follow-up email should be sent promptly, typically within one or two days after the interview. Following this email, give the professor or the admissions committee enough time to interview other candidates and make their final decisions. If you don’t hear back for two or three weeks, it is okay to send a short follow-up message asking about their decision timeline. Remember to be polite and professional with all of your communications. Keep in mind that regardless of the decision, you may interact with people you interviewed with in the future.

## Rule 10: Imagine the future

The interview is a unique opportunity to interact with the people that could possibly be a part of your life for the next few years, as well as an opportunity to learn more about the institution and the city where you will be working and living. However, when it comes to making the actual decision about where you will study or work, it can often be challenging. This is particularly true in an era of video interviews, when you might not have the opportunity to interact with students, professors, or the surrounding neighborhood. Given the limitations of virtual interviews, visiting the campus or surrounding area will help establish context when talking to people about the environment. If the university offers a “second look” video tour or an opportunity to email or chat with students prior to accepting an offer of admission, take the time to learn more about the choices you are facing. As mentioned in Rules 4 and 8, even if the school does not offer a formal program, do not be afraid to email current trainees to ask the tough questions.

## Conclusion: Good luck and have fun!

The interview is a unique opportunity to interact with the people, the institution, and the city that may be a part of your life for the next few years. Due to the COVID-19 pandemic, the way in which we do science and conduct interviews has changed overnight. Some of the practices that were useful for in-person interviews may no longer apply or will need to be adapted to the new “normal.” While we look forward to resume in-person activities, virtual interviews provide unique opportunities to reduce environmental impact and increase the diversity of applicants. Therefore, virtual interviews may become part of the “new normal.” For interviewees interested in other supplemental resources, we would encourage you to browse the home page of the National Institutes of Health (NIH’s) Office of Intramural Training and Education (OITE) for resources under “trainees outside the NIH” or the OITE YouTube page: https://www.youtube.com/c/NIHOITE. In addition, for interviewees interested in advice on how to choose their next steps, there are resources to help select potential advisors or schools at https://www.training.nih.gov/mentoring_guidelines. While virtual interviews raise unique challenges, they also present interesting opportunities. We hope these rules will provide younger trainees a way to feel comfortable with the new interview format and assist them with making an informed choice about their future.
